# Synergistic effects of EMPs and PMPs on pulmonary vascular leakage and lung injury after ischemia/reperfusion

**DOI:** 10.1186/s12964-020-00672-0

**Published:** 2020-11-23

**Authors:** Jie Zhang, Yu Zhu, Yue Wu, Qing-Guang Yan, Xiao-Yong Peng, Xin-Ming Xiang, Ming-Ying Xue, Qing-Hui Li, Liang-Ming Liu, Tao Li

**Affiliations:** State Key Laboratory of Trauma, Burns and Combined Injury, Shock and Transfusion Department of Research Institute of Surgery, Daping Hospital, Third Military Medical University, Army Medical University, Daping, Chongqing, 400042 People’s Republic of China

**Keywords:** Emps, Pmps, Pulmonary vascular permeability

## Abstract

**Background:**

Vascular leakage is an important pathophysiological process of critical conditions such as shock and ischemia–reperfusion (I/R)-induced lung injury. Microparticles (MPs), including endothelial cell-derived microparticles (EMPs), platelet-derived microparticles (PMPs) and leukocyte-derived microparticles (LMPs), have been shown to participate in many diseases. Whether and which of these MPs take part in pulmonary vascular leakage and lung injury after I/R and whether these MPs have synergistic effect and the underlying mechanism are not known.

**Methods:**

Using hemorrhage/transfusion (Hemo/Trans) and aorta abdominalis occlusion-induced I/R rat models, the role of EMPs, PMPs and LMPs and the mechanisms in pulmonary vascular leakage and lung injury were observed.

**Results:**

The concentrations of EMPs, PMPs and LMPs were significantly increased after I/R. Intravenous administration of EMPs and PMPs but not LMPs induced pulmonary vascular leakage and lung injury. Furthermore, EMPs induced pulmonary sequestration of platelets and promoted more PMPs production, and played a synergistic effect on pulmonary vascular leakage. MiR-1, miR-155 and miR-542 in EMPs, and miR-126 and miR-29 in PMPs, were significantly increased after hypoxia/reoxygenation (H/R). Of which, inhibition of miR-155 in EMPs and miR-126 in PMPs alleviated the detrimental effects of EMPs and PMPs on vascular barrier function and lung injury. Overexpression of miR-155 in EMPs down-regulated the expression of tight junction related proteins such as ZO-1 and claudin-5, while overexpression of miR-126 up-regulated the expression of caveolin-1 (Cav-1), the trans-cellular transportation related protein such as caveolin-1 (Cav-1). Inhibiting EMPs and PMPs production with blebbistatin (BLE) and amitriptyline (AMI) alleviated I/R induced pulmonary vascular leakage and lung injury.

**Conclusions:**

EMPs and PMPs contribute to the pulmonary vascular leakage and lung injury after I/R. EMPs mediate pulmonary sequestration of platelets, producing more PMPs to play synergistic effect. Mechanically, EMPs carrying miR-155 that down-regulates ZO-1 and claudin-5 and PMPs carrying miR-126 that up-regulates Cav-1, synergistically mediate pulmonary vascular leakage and lung injury after I/R.

**Graphic abstract:**

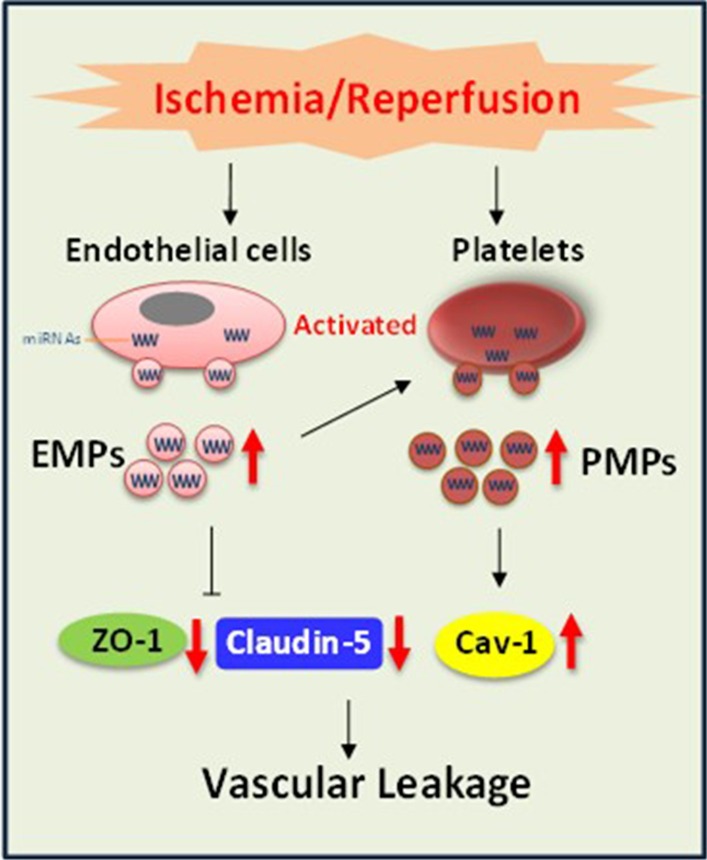

**Video Abstract**

## Background

Ischemia/reperfusion (I/R)-induced lung injury, which further leads to respiratory failure and even death, is commonly seen in cardiopulmonary resuscitation, lung transplantation and shock-resuscitation. Vascular leakage is a key pathophysiological process of I/R-induced lung injury. The mechanism mainly includes para-cellular pathway and trans-cellular pathway. The para-cellular pathway is mainly related to the destruction of tight junctions and adhesion junctions and the degradation of their major constituent proteins, such as ZO-1, claudin-5 and VE-Cadherin (VE-Cad). The trans-cellular pathway is mainly related to the activation of cavealin-1 (Cav-1). Currently, many treatments based on current mechanisms can not effectively improve the vascular leakage [1, 2], suggesting that the mechanism underlying vascular leakage is not entirely understood.

Microparticles (MPs) are a group of small vesicles with the diameter of 100 nm–1 µm, which are produced by a variety of cells after activation and apoptosis. In recent years, MPs have been greatly paid attention because of their pathogenic effects in many diseases. For example, platelet-derived MPs (PMPs) may lead to tubular epithelial cell apoptosis and renal injury after I/R [3]. Endothelial cell-derived MPs (EMPs) plays an important role in carbon tetrachloride (CCl4) induced hepatic fibrosis [4]. Recently, the pathogenic role of MPs in lung injury has also been studied [5], for instance, red cell-derived microparticles (RMPs) are potential mediators of transfusion-related acute lung injury [6, 7]. However, those studies only observed the role of one kind of MPs in tissue and organ injury. Moreover, I/R may impose detrimental effects on serious of cells, including endothelial cells, platelets and leukocytes, probably to induce MPs production [8–10]. Thus, whether endothelial cell-derived MPs (EMPs), platelet-derived MPs (PMPs) and leukocyte-derived MPs (LMPs) are increased after I/R, and which one takes part in pulmonary vascular leakage, and if they have synergetic effect and the underlying mechanism are unclear.

Studies have shown that pro-inflammatory cytokines, chemokines, miRNAs and DNAs are the major materials packaged in MPs, among which miRNAs have been widely studied [11]. MiRNAs packaged in MPs play a vital role in many pathological processes, for example, MPs can carry miRNA-128-3p to hepatic stellate cells, modulating the process of liver fibrosis [12]. Whether MPs regulation of vascular permeability is related to miRNAs is not known.

Thus, using I/R rat model and H/R pulmonary vascular endothelial cells (VECs), we investigated the role and mechanisms of EMPs, PMPs and LMPs in pulmonary vascular leakage, and the mechanisms.

## Materials and methods

### Experimental animals

The present study conformed to the principles of the “Guide for the Care and Use of Laboratory Animals” (eighth edition, 2011, Washington, DC, National Academies Press, USA) and was approved by the Laboratory Animal Welfare and Ethics Committee of the Third Military Medical University, Chongqing, People’s Republic of China (Ethic Approval: 2017042201).

Sprague–Dawley rats (200–220 g) were purchased from the Animal Center of Research Institute of Surgery, Third Military Medical University (Army Medical University). The animal management for I/R and hemorrhage-resuscitation was described as follow. The rats were anesthetized with sodium pentobarbital (initial dose: 30 mg/kg, intraperitoneal). The right femoral artery was catheterized with poly-ethylene catheters to monitor the mean arterial pressure (MAP) and bleeding, and the right femoral vein was subjected to the same management if drug administration was needed.

For hemorrhage-transfusion model, the rats were hemorrhaged 50% blood of total blood volume through the right femoral arterial catheter within an hour and then maintained for another 30 min. Afterward, the same volume of whole blood was transfused, and then the rat was maintained for another 2 h for experiments.

For I/R model, the rats were prepared as follows: the aorta abdominalis was occluded using a microvascular clamp for 1 h, and then the clamp was removed to allow reperfusion for 3 h.

### Isolation of MPs from blood and cultured endothelial cells, leukocytes and platelets

Differential centrifugation was used for MP isolation as described by Fink [13], Jansen [14], and Fujii [15]. For isolation of whole blood MPs, platelet-free plasma was first obtained from 5 mL of whole blood by centrifugation at 2000 g for 40 min and was then subjected to centrifugation at 20,000 g for 40 min. The sediments were suspended in 100 μL of PBS and stored at − 80 °C. For isolation of EMPs, LMPs and PMPs, 4 mL of culture medium from endothelial cells, leukocytes and platelets were subjected to centrifugation at 2000 g for 40 min, and then the supernatant was subjected to further centrifugation at 200,000 g for 40 min. The sediments were suspended in 100 μL of PBS and stored at − 80 °C. The MP content was measured by BCA protein assays (Thermo Scientific, USA) and then adjusted to the same concentration for further experiments.

### Quantitative determination of MPs by flow cytometry and ELISA

All of the MP samples were washed with PBS and then centrifuged at 20,000 g for 40 min. The sediments were suspended in 100 μL of PBS containing 10 μL of 10 × Annexin V binding buffer (10 mM HEPES, pH 7.4, 140 mM NaCl, and 2.5 mM CaCl_2_) and stained with the following 5 μM antibodies: PerCP-Cy7-CD31 and PE-CD144 for EMPs, APC-CD45 for LMPs, PE-CD61 for PMPs, and FITC-Annexin V whole plasma MPs. All samples were maintained at room temperature for 30 min in the dark. Then, Image Stream X Mk II (Amnis Corporation, Seattle, USA) was used to analyze the MP profiles as described by Headland [16]. In addition, quantitative determination of MPs was done by ELISA as described by Hellum [17]. The Zymuphen MP activity assay (Hyphen BioMed, Neuville-sur-Oise, France) was used to determine the activity of phosphatidylserine (PS), which widely exists on the membrane surface of MPs. Briefly, 10 μL of MPs was added to an ELISA plate coated with Annexin V-streptavidin for 1 h at 37 °C. Then, 100 μL of R1 solution, 50 μL of R2 solution and 50 μL of R3 solution were dispensed in sequence for preincubation at 37 °C for 15 min. Finally, 50 μL of stop solution was added, and Synergy HT (BioTek, Winooski, VT, USA) was used to read the OD at 405 nm to determine the MP content.

Other materials and methods, including *Measurement of the permeability of the pulmonary vasculature and endothelial cell monolayers, Histological analysis and immunofluorescence, Identification of MPs, Transfection of double-stranded RNA (dsRNA)* and so on, are illustrated in Additional file 1.

## Results

### MPs play an important role in pulmonary vascular leakage and lung injury after I/R

**Fig. 1:**
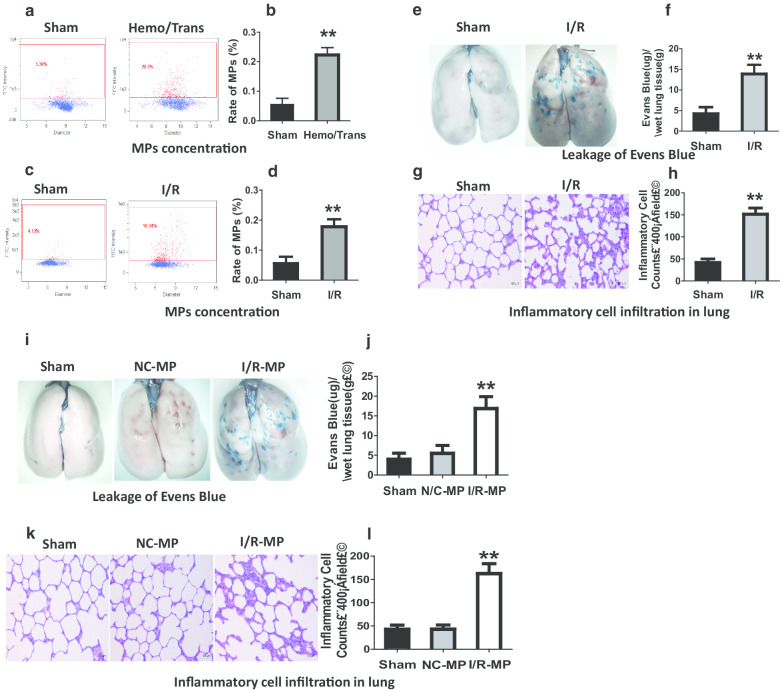
The role of MPs in pulmonary vascular leakage and lung injury after I/R. **a**–**d** The change of MP concentration in blood in hemorrhage-transfusion model (Hemo-Trans) (**a**, **b**) and ischemia/reperfusion model (I/R) (**c**, **d**), MPs were marked with annexin V antibodies. **e**, **f** The change of pulmonary vascular permeability reflected by leakage of Evans blue 30 min after injection (iv) in I/R rats. **g**, **h** The status of inflammatory cells infiltration in lung after I/R. The numbers of inflammatory cells were determined in 10 randomly selected nonoverlapping fields at 400 × magnification in respective sections of the individual rat lung. **i**, **j** Effects of intravenous administration of NC-MPs and I/R-MPs on pulmonary vascular permeability reflected by the leakage of Evans blue. **k**, **l** Effects of NC-MPs and I/R-MPs on inflammatory cells infiltration in lung. The data are the mean ± SD of n experiments (n = 8). *Sham* sham-operated group, *NC-MP* MPs derived from the blood of normal rats; *I/R-MP* MPs derived from the blood of ischemia/reperfusion rats. **P < 0.01 versus N or sham group

To explore the relationship between MPs and vascular permeability, I/R and hemorrhage-transfusion (Hemo/Trans) rats were utilized, and flow cytometry was used to analyze MP concentrations in blood. The results showed that the concentration of MP in blood was significantly increased in Hemo/Trans and I/R rats as compared to sham groups (Fig. 1a–d). Simultaneously, pulmonary vascular permeability was increased (Fig. 1e, f). In addition, large amount of inflammatory cells leaked into the interstitial space of lung (Fig. 1g, h) and the protein concentration in bronchoalveolar lavage fluid (BALF) was significantly increased in I/R rats (Additional file 2: Fig. S1B). Furthermore, H/R-treated pulmonary VECs were used to confirm the relationship between MPs and vascular permeability. The results showed that H/R induced MP production (Additional file 2: Fig. S1C) and caused the increase of monolayer pulmonary VECs permeability with the decrease of trans-endothelial resistance (TER) of monolayer pulmonary VECs (Additional file 2: Fig. S1D). The results showed that the the change of MP concentration in blood is positively related to the change of vascular permeability after I/R.

To evaluate the role of MPs in pulmonary vascular leakage and lung injury after I/R, MPs from normal rats (NC-MPs) and I/R rats (I/R-MPs) were obtained and administered to the normal rats (1 × 10^8^/kg, iv), then the pulmonary vascular leakage and lung injury were observed. The results showed that I/R-MPs induced Evans blue and FITC-labeled albumin leakage to lung tissue, while NC-MPs had no effect (Fig. 1i, j, Additional file 2: Fig. S1E). In the microvasculature, I/R-MPs also induced the leakage of FITC-labeled albumin into the extra-vascular space (Additional file 2: Fig. S1F). Additionally, I/R-MPs induced inflammatory cells infiltration to lung tissue and led to interstitial edema (Fig. 1k, l) and also increased the protein concentration in BALF (Additional file 2: Fig. S1G). Moreover, in vitro I/R-MPs decreased the TER of monolayer pulmonary VECs (Additional file 2: Fig. S1H). These results demonstrated that MPs played a vital role in pulmonary vascular leakage and lung injury after I/R.

### EMPs and PMPs have synergistic effect on pulmonary vascular leakage and lung injury after I/R.

**Fig. 2:**
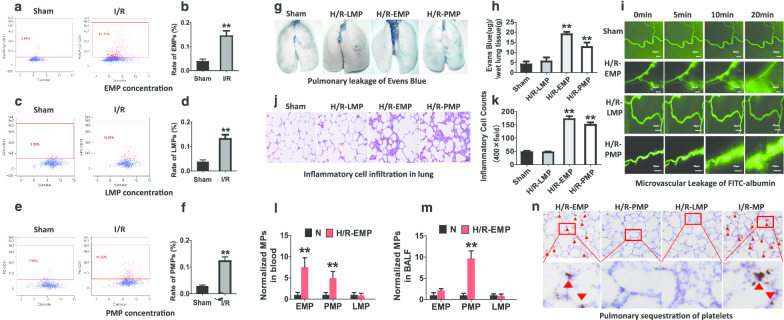
The changes of EMPs, PMPs and LMPs in blood after I/R and their role in pulmonary vascular hyperpermeability and lung injury. **a**, **b** The change of EMPs in blood marked with CD31 antibodies. **c**, **d** The change of LMPs in blood marked with CD45 antibodies. **e**, **f** The change of PMPs in blood marked with CD61 antibodies. **g**, **h** Effects of H/R-EMPs, H/R-PMPs and H/R-LMPs on pulmonary vascular permeability reflected by Evans Blue leakage. **i** The effect of H/R-EMPs, H/R-PMPs and H/R-LMPs on leakage of FITC (fluorescein isothiocyanate)-labeled albumin in microvasculature. **j**, **k** The status of inflammatory cells infiltration in lung 2 h after intravenous administration of H/R-EMPs, H/R-PMPs and H/R-LMPs. The numbers of inflammatory cells were determined in 10 randomly selected nonoverlapping fields at 400 × magnification in respective sections of the individual rat lung. **l**, **m** The effects of H/R-EMPs on the concentrations of EMPs, PMPs and LMPs in plasma (**l**) and BALF (bronchoalveolar lavage fluid) (**m**). **n** The effects of H/R-EMPs, H/R-PMPs and H/R-LMPs on pulmonary sequestration of platelets by immunohistochemical staining, CD41 antibodies were used to mark platelets. The data are the mean ± SD of n experiments (n = 8). NC-LMP, NC-EMP, NC-PMP: derived from normal leukocytes, vascular endothelial cells and platelets, respectively; H/R-LMP, H/R-EMP, H/R-PMP: derived from hypoxia/reoxygenation-treated leukocytes, vascular endothelial cells and platelets, respectively. **P < 0.01 versus N or sham group

Blood MPs can be derived from platelets, red blood cells, endothelial cells and leukocytes. But it is not known which one takes part in vascular leakage after I/R. Thus, we firstly observed the change of EMPs, LMPs and PMPs in blood after I/R. The results indicated that the concentrations of EMPs (Fig. 2a, b, Additional file 3: Fig. S2A, B), LMPs (Fig. 2c, d) and PMPs (Fig. 2e, f) in blood were significantly increased after I/R. In vitro, H/R stimulation also induced EMP, LMP and PMP production in VECs, leukocytes and platelets respectively (Additional file 3: Fig. S2C). Further, administration of H/R-PMPs and H/R-EMPs (Produced in H/R treated platelets and VECs) significantly increased the vascular permeability of normal rats, including a striking pulmonary vascular leakage (Fig. 2g, h) and microvasculature leakage (Fig. 2i), while H/R-LMPs had no effect. Additionally, H/R-PMPs and H/R-EMPs also induced inflammatory cells infiltration into the pulmonary interstitial space (Fig. 2j, k) and increased the protein concentration in BALF (Additional file 3: Fig. S2D). In vitro, H/R-PMP and H/R-EMP stimulation led to the decrease of the TER of monolayer pulmonary VECs (Additional file 3: Fig. S2E). These results suggest that EMPs and PMPs are the main MPs that participate in pulmonary vascular leakage and lung injury after I/R.

To evaluate the synergistic effect of EMPs and PMPs in the regulation of pulmonary permeability, EMPs and PMPs were infused to the normal rats through the veins, and then the concentrations of the MPs in blood and BALF were analyzed. The results showed that H/R-EMPs increased EMPs in blood as expected, and meanwhile the PMPs both in blood and BALF were also increased (Fig. 2l, m). And interestingly, the PMPs in BALF was higher than that in blood (Fig. 2l, m). Inversely, H/R-PMPs only increased PMPs in blood and BALF but not EMPs or LMPs in blood and BALF (Additional file 3: Fig. S2F, G). These results suggest that H/R-EMPs can induce PMPs production. The further study found that H/R-EMPs induced obvious pulmonary sequestration of platelets, while H/R-PMPs and H/R-LMPs did not had such effect (Fig. 2n). These results suggested that in addition to directly regulating pulmonary vascular permeability, EMPs may exacerbate pulmonary leakage and lung injury after I/R through inducing pulmonary sequestration of platelets and PMP production, EMPs and PMPs play a synergistic effect on pulmonary vascular leakage and lung injury.

### EMPs and PMPs transport miR-155 and miR-126 respectively to regulate pulmonary vascular permeability.

**Fig. 3:**
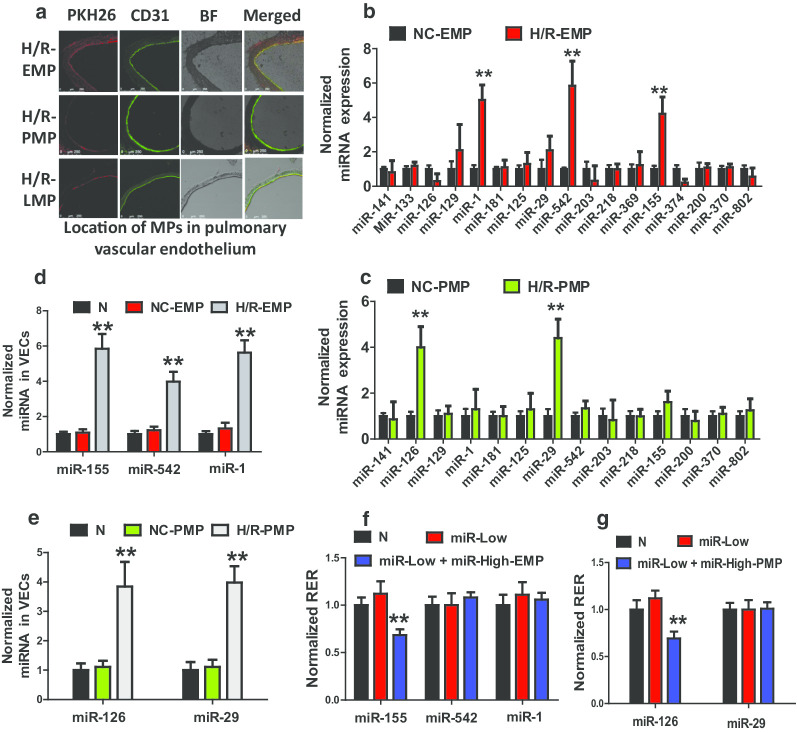
The role of miRNAs in EMPs and PMPs in pulmonary vascular leakage and lung injury. **a** The localization of H/R-LMPs, H/R-EMPs, and H/R-PMPs in the pulmonary vascular endothelial cells after intravenous administration. The vascular endothelial cell was stained with CD31 antibodies (green), and MPs were stained with PKH26 (red). **b** Level of miRNAs related to vascular permeability in NC-EMPs and H/R-EMPs. **c** The level of miRNAs related to vascular permeability in NC-PMPs and H/R-PMPs. **d** Level of miRNAs in VECs 1 h after NC-EMPs and H/R-EMPs treatment. **e** Level of miRNAs in VECs 1 h after NC-PMPs and H/R-PMPs treatment. **f** Effect of miRNAs transported by EMPs on the TER of monolayer VECs. **g** Effect of miRNAs transported by PMPs on the TER of monolayer VECs. Data are the mean ± SD of n experiments (n = 8). *miR-Low*: miRNA low expression; *miR-High-EMP*: miRNA-high containing EMP; *miR-High-PMP*: miRNA-high containing PMP. **P < 0.01 versus N groups or NC-EMP groups or NC-PMP groups

To confirm the direct interaction between MPs and VECs, PKH26-labeled EMPs, PMPs and LMPs were administered intravenously to normal rats. The results showed that PKH26-labeled EMPs, PMPs and LMPs massively located in the endothelium of the pulmonary vasculature (Fig. 3a) and microvasculature (Additional file 4: Fig. S2A). In addition, in vitro the EMPs, PMPs and LMPs were absorbed by pulmonary VECs after coculture for 30 min (Additional file 4: Fig. S3B, C).

To investigate the mechanism of EMP and PMP regulation of vascular permeability, 17 miRNAs that are closely related to vascular permeability were screened, including miR-141, miR-133, miR-126, miR-129, miR-1, miR-181, miR-125 and so on. The results showed that the amount of miR-1, miR-155, and miR-542 in H/R-EMP were significantly higher than those in NC-EMP (Fig. 3b), while the amount of miR-29 and miR-126 in H/R-PMP were significantly higher than those in NC-PMP (Fig. 3c). Further experiments showed that in H/R-EMP treated pulmonary VECs, the expression of miR-1, miR-155 and miR-542 was increased (Fig. 3d), while in H/R-PMPs treated VECs, the expression of miR-126 and miR-29 was increased (Fig. 3e), These results suggested that H/R-EMPs and H/R-PMPs may transport different miRNAs to pulmonary VECs to regulate permeability. To confirm this result, red fluorescence-labeled miR-155 was transfected into the pulmonary VECs, which were then used to produce EMPs. The EMPs were cocultured with VECs for 30 min. Expectedly, The red fluorescence labeled miR-155 was observed entering into the VECs (Additional file 4: Fig. S3D), demonstrating that MPs transport miRNAs to the target cells.

To unveil the role of miRNAs in EMPs and PMPs in regulation of vascular permeability, high and low levels of miRNAs containing EMPs and PMPs were used to stimulate corresponding miRNA-inhibited monolayer pulmonary VECs. The results showed that inhibition of miR-1, miR-29, miR-126, miR-155 and miR-542 had no effects on the TER of monolayer VECs, as compared to the normal groups; however, miR-155-high containing EMPs and miR-126-high containing PMPs significantly decreased the TER of monolayer pulmonary VECs (Fig. 3f, g). The other miRNA-high-containing EMPs and PMPs had no influence on TER of pulmonary VEC (Fig. 3f, g). These results suggest that EMPs carrying miR-155 and PMPs carrying miR-126 participate in regulation of vascular permeability.

### EMPs transporting miR-155 that targets ZO-1 and claudin-5, and PMPs transporting miR-126 that targets Cav-1, synergistically regulate pulmonary vascular permeability after I/R

**Fig. 4:**
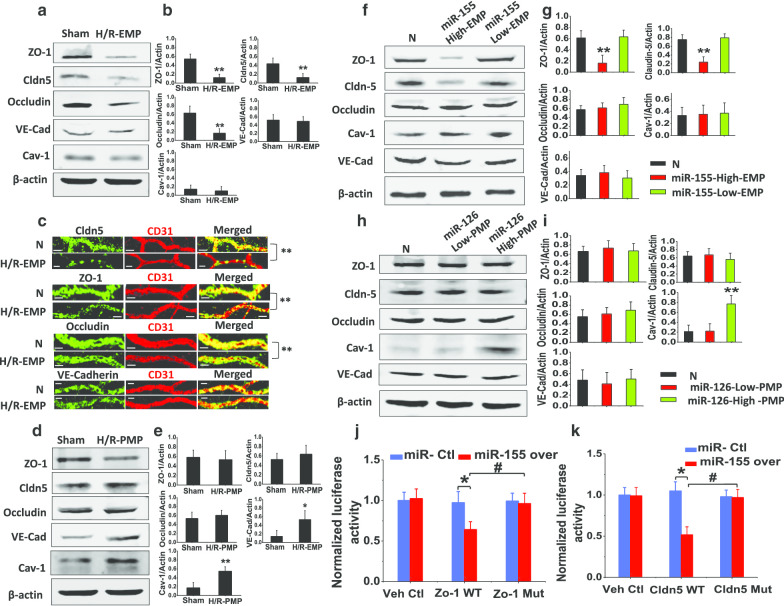
The mechanism of miR-155 and miR-126 in H/R-EMP and H/R-PMP induced vascular leakage. **a**, **b** Effect of H/R-EMPs on the expression of vascular permeability-related proteins in pulmonary vasculature of rats. **c** Effect of H/R-EMPs on vascular permeability-related proteins in microvasculature of rats (scale bars 25 μm). **d**, **e** Effect of H/R-PMPs on the expression of vascular permeability-related proteins in pulmonary vasculature of rats. **f**, **g** Effect of miR-155 high and low containing EMPs on the expression of vascular permeability-related proteins in VECs. **h**, **i** Effect of miR-126 high and low containing PMPs on the expression of vascular permeability-related proteins in VECs. **j**, **k** Effect of miR-155 on the transcription of ZO-1 and claudin-5 as measured by a dual-luciferase reporter system. Data are mean ± SD of n experiments (n = 8). *Veh Ctl* vehicle control, *WT* wild type; *Mut* mutant type. *P < 0.05 miR-155 over versus miR-Ctl in ZO-1 and Cldn-5 WT group; **P < 0.01 versus normal group; #*P* < 0.05 ZO-1 WT group versus ZO-1 Mut group with miR-155 over, or cldn-5 WT group versus cldn-5–1 Mut group with miR-155 over

To investigate how miR-155 transported by EMPs and miR-126 transported by PMPs regulate pulmonary vascular permeability, the effects of H/R-EMPs and H/R-PMPs on the expression of ZO-1, cluadin-5, occludin and VE-cad, and Cav-1 were observed both in vivo and in vitro. The results showed that intravenous administration of H/R-EMPs significantly inhibited the expression of ZO-1, claudin-5 and occludin in the pulmonary vasculature (Fig. 4a, b). Moreover, H/R-EMPs inhibited the expression of ZO-1, claudin-5 and occludin in the microvasculature (Fig. 4c). These results were confirmed by western blot and confocal microscopy imaging in vitro in pulmonary VECs (Additional file 5: Fig. S4A–D). H/R-PMPs induced the high expression of Cav-1 and VE-cad in pulmonary vasculature of rats (Fig. 4d, e) and also in pulmonary VECs, H/R-PMP only induced the expression of Cav-1 (Additional file 5: Fig. S4E).

Furthermore, the effects of miR-155-high containing EMPs and miR-126-high containing PMPs on the expression of vascular permeability proteins were observed. The results showed that miR-155-highcontaining EMPs significantly decreased the expression of ZO-1 and claudin-5 (Fig. 4f, g) but had no effect on occludin, Cav-1 or VE-Cad. MiR-126-high containing PMPs increased the expression of Cav-1 but had no effect on ZO-1, claudin-5, occludin, or VE-Cad (Fig. 4h, i). These results suggest that miR-155 decreases the expression of ZO-1 and claudin-5, miR-126 increases the expression of Cav-1. To elucidate the mechanism by which miR-155 regulates the expression of ZO-1 and claudin-5, a dual-luciferase reporter system was used. The results showed that upregulation of miR-155 significantly reduced the translation of target gene (Fig. 4j, k). These results suggest that EMPs transporting miR-155 to down-regulate ZO-1 and claudin-5 and PMPs transporting miR-126 to up-regulate Cav-1 synergistically induce pulmonary vascular leakage via both para-cellular and trans-cellular pathway.

### Inhibition of EMP and PMP production benefits pulmonary vascular permeability and lung injury after I/R

**Fig. 5:**
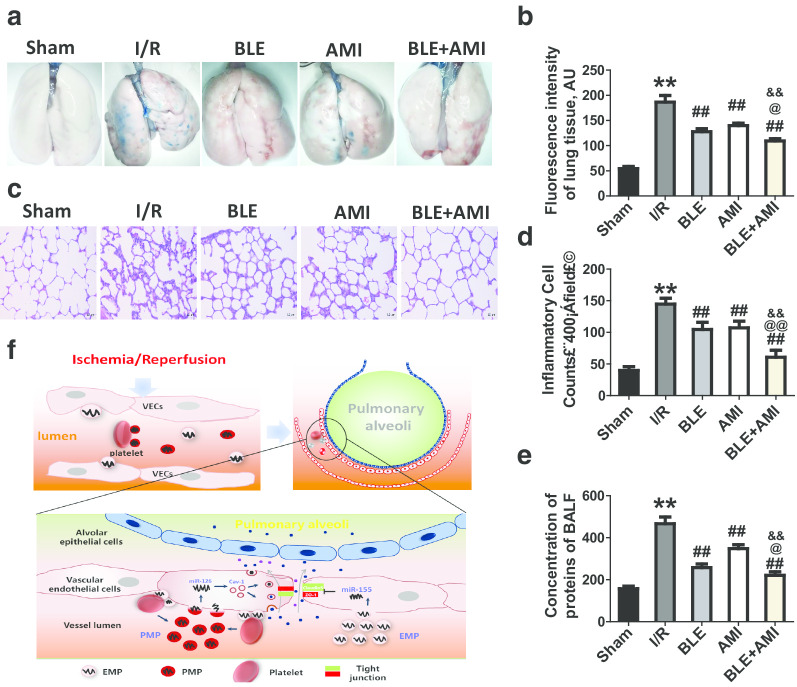
The beneficial effect of blebbistatin and amitriptyline on vascular leakage and lung injury after I/R in rats. **a** Beneficial effect of EMP inhibitor BLE (blebbistatin) and PMP inhibitor AMI (amitriptyline) on pulmonary vascular permeability reflected by leakage of Evan Blue. **b** Effect of BLE and AMI alone, and their combined use on pulmonary vascular permeability reflected by leakage of FITC-labeled albumin. **c**, **d** Beneficial effect of BLE (10 mg/kg) and AMI (5 mg/kg)on pulmonary inflammation of I/R rats. The numbers of inflammatory cells were determined in 10 randomly selected nonoverlapping fields at 400 × magnification in respective sections of the individual rat lung. **e** Beneficial effect of BLE and AMI on BALF proteins after I/R. **f** Schematic diagram of the mechanism by which EMPs mediate pulmonary sequestration of platelets and PMP production and EMPs and PMPs regulate pulmonary vascular permeability. The data are the mean ± SD of n experiments (n = 8). **P < 0.01 versus the sham group; ##*P* < 0.01 versus the I/R group; @*P* < 0.05, @@*P* < 0.01 versus the BLE group; &&*P* < 0.01 versus the AMI group

To verify the role of EMPs and PMPs in pulmonary vascular leakage and lung injury after I/R, the effect of EMP inhibitor blebbistatin (BLE) [18] and PMP inhibitor amitriptyline (AMI) [19] on pulmonary vascular permeability and lung injury were observed. The results showed that BLE (10 mg/kg, iv) and AMI (10 mg/kg, iv) markedly reduced the leakage of Evans blues and FITC-labeled albumin into pulmonary interstitial space and improved vascular permeability (Fig. 5a, b). Moreover, BLE and AMI treatment significantly alleviated inflammatory cells infiltration (Fig. 5c, d) and reduced the concentration of proteins in BALF (Fig. 5e). Notably, the combined treatment of BLE (10 mg/kg) and AMI (5 mg/kg) significantly improved pulmonary vascular permeability and lung injury (Fig. 5a–e), exhibiting a good synergistic effect. These results suggest that inhibition of EMP and PMP production benefits pulmonary vascular permeability and lung injury and may be a potential therapeutic strategy.

## Discussion

The present study showed that EMPs and PMPs in blood led to pulmonary vascular leakage and lung injury after I/R. In addition to directly imposing a detrimental effect on the pulmonary vasculature, EMPs induced the pulmonary sequestration of platelets to produce PMPs, EMPs and PMPs synergistically induced pulmonary vascular leakage and lung injury. EMP transported miR-155 to target ZO-1 and claudin-5 and PMPs transported miR-126 to up-regulate caveolin-1, synergistically inducing pulmonary vascular leakage through para-cellular and trans-cellular pathways, respectively, after I/R (Fig. 5f).

The role of MPs in diseases has attracted considerable attention in recent years. The production of MPs in blood is closely related to the progression of many diseases. For instance, MPs mediates hepatic I/R injury through activation of inflammatory signal pathway [20]. In addition, the role of MPs in vascular barrier function has also been studied. In the condition of ischemia, EMPs increased the permeability of microvascular endothelial barriers [21]. However, these studies merely focused on one kind of MPs to observe its effect, also did not observe the comprehensive effects of multiple MPs. Our present study found that EMPs and PMPs participated in the regulation of pulmonary vascular permeability and lung injury after I/R. EMPs and PMPs played synergistic effect. In addition to directly inducing pulmonary vascular leakage, EMPs mediated the platelets sequestration in the lung, and induced more PMPs production to synergistically induce the vascular leakage. But how EMP mediates the platelets sequestration and induces PMP production were not investigated in present study, it needs further study. Previous studies showed that platelets sequestration was dependent on the interaction between the platelets and endothelial cells via ligands, such as collagen, fibrinogen and intercellular adhersion molecules (ICAM) on the surface of platelets and endothelial cells [22, 23]. The membrane of EMPs inherits serious of proteins from VECs, including integrin and platelet adhesion molecules, which can mediate endothelial adhesion of platelets under physiological conditions. Thus, we speculated that the mechanism by which EMPs mediated pulmonary sequestration of platelets may be related to these membrane proteins. In addition, platelets activation and consequent PMP production is dependent on the increase of cytoplasmic calcium and cytoskeleton reorganization [24, 25]. Cauwenberghs S et al. found that MP formation required the αIIbβ3 signaling to destabilization of the actin cytoskeleton [26]. Meanwhile, one study found that VEC-derived protein disulfide isomerase could mediate GP IIb/IIIa receptor activation and induced PMP production [25]. Whether EMP inherits disulfide isomerase from endothelial cells, and by which EMP inducing the PMP release needs further investigation.

EMPs and PMPs contain a large number of active substances from the parent cells, such as proteins, lipids, DNA and miRNAs. MiRNAs are short noncoding RNAs that encode approximately 30% of the genes of a cell and have multiple biological functions [27]. Studies have showed that the role of miRNAs in pathogenic function of MPs [28]. For instance, epithelial deprived MPs carrying miR-17/221 modulates macrophage β integrin recycling, promoting macrophage recruitment and ultimately contributing to lung inflammation [29]. Whether miRNAs participate in synergistic effect of EMP and PMP regulation of pulmonary vascular permeability and which miRNA is related? In the present study, we found that miR-155 played important role in EMP regulation of pulmonary vascular permeability and that miR-126 participated in PMP regulation of pulmonary vascular permeability. In addition, miR-1 and miR-542 were transported by EMPs and miR-29 was transported by PMPs to pulmonary VECs. Although, previous reports showed that miR-1 and miR-29 played important role in regulating vascular endothelial barrier function [30, 31]. In present study, however, miR-1, miR-542 and miR-29 had no effect on vascular permeability. We speculate that the main reason for this disparity is that the absolute level of miR-1, miR-542 and miR-29 in EMPs and PMPs is low. Further studies are needed.

Present study demonstrated that miR-155 in EMP down-regulated the expression of ZO-1 and claudin-5 and miR-126 in PMP up-regulated the expression of Cav-1. ZO-1 and claudin-5 are the major constituent proteins of tight junction. The decrease of ZO-1 and claudin-5 induced destruction of tight junctions and led to vascular leakage through the para-cellular pathway; Cav-1 is a major protein of caveolae, which mediates the trans-cellular transportation of macromolecule. The present study suggests that EMPs transport miR-155 that targets ZO-1 and claudin-5, and PMPs transport miR-126 that targets caveolin-1, synergistically regulating pulmonary vascular permeability after I/R.

Ultimately, we observed the effect of inhibiting MPs production on pulmonary vascular permeability and lung injury and found that EMP inhibitor blebbistatin (BLE) and PMP inhibitor amitriptyline (AMI) reduced pulmonary vascular permeability and alleviated lung injury in I/R rats, showing that inhibition of MPs production may be a potential therapeutic strategy for vascular leakage after I/R injury. BLE is a nonmyosin II-regulated light-chain (RLC) phosphorylation inhibitor, which can inhibit the formation and release of EMP by blocking the myosin heavy chain binding to actin and submembranous location [18]. AMI is an FDA-approved antidepressant and sphingolipase inhibitor, which has been shown to inhibit MP production, possibly by inhibiting the acid sphingolipase [32]. Based on literature and our pilot study, 10 mg/kg BLE and AMI (dissolved in sterile saline) were used in the present study. Awojoodu AO et al. observed effect of AMI on MPs production at four concentrations (0, 2.5, 5, 10 km/mg) in mice, and found that 10 mg/kg AMI significantly inhibited the MPs production [19]. In the present study, 10 mg/kg AMI alone exhibited a beneficial effect on vascular permeability, however when combined with BLE, 5 mg/kg AMI plus BLE showed a better synergistical effect. Our preliminary experiments observed the effect of 1, 5, 10 and 20 mg/kg BLE on MPs production and animal survival for I/R rats, the results showed that 10 mg/kg of BLE exhibited better effect on the inhibition of MPs production and animal survival in I/R rats (data not showed).

The limitations of this study mainly include: First, whether other MPs such as erythrocyte-derived MPs, macrophage-derived MPs and alveolar epithelial cell-derived MPs, are involved in the regulation of pulmonary vascular permeability after I/R have not been studied. Second, the present study only investigated the role of miRNAs in MP-mediated regulation of pulmonary vascular permeability and lung injury; whether other substances in MPs such as DNA and circRNAs can be transported to target cells to play effects have not been investigated. Third, the mechanisms by which EMP mediates platelets sequestration and PMP release is not studied.

## Conclusion

EMP and PMP play an important role in increased pulmonary vascular hyperpermeability and lung injury after I/R; EMPs may promote pulmonary sequestration of platelets and PMP production to play synergistic effect; EMPs may transport miR-155 to target ZO-1 and claudin-5 and PMPs transport miR-126 to up-regulate caveolin-1, synergistically resulting in pulmonary vascular leakage and lung injury through para-cellular and trans-cellular pathways after I/R. The study not only provided a new target for the potential treatment of lung injury but also provided a new strategy to protect against lung injury after I/R.

## Supplementary information

**Additional file 1**. Supplemental Digital Content.

**Additional file 2**. Supplemental Figure 1.

**Additional file 3**. Supplemental Figure 2.

**Additional file 4**. Supplemental Figure 3.

**Additional file 5**. Supplemental Figure 4.

## Data Availability

All raw data are available on request.

## Abbreviations

MPs: Microparticles
EMPs: Endothelial cell-derived microparticles
PMPs: Platelet-derived microparticles
LMPs: Leukocyte-derived microparticles
I/R: Ischemia/reperfusion
Hemo/Trans: Hemorrhage/transfusion
H/R: Hypoxia/reoxygenation
BALF: Bronchoalveolar Lavage Fluid
NC-MPs: MPs from normal rats
I/R-MPs: MPs from I/R rats
TER: Trans-endothelial electrical resistance
VEC: Vascular endothelial cells
Cav-1: Cavleolin-1
BLE: Blebbistatin
AMI: Amitriptyline
