# Supplementary material for: Synergistic effects of EMPs and PMPs on pulmonary vascular leakage and lung injury after ischemia/reperfusion
Source: Cell Commun Signal. 2020 Nov 23;18:184. doi: 10.1186/s12964-020-00672-0 (PMC7682096; doi:10.1186/s12964-020-00672-0)
Supplement: Supplementary file 2 — Additional file 1. Supplemental Digital Content. [file 12964_2020_672_MOESM2_ESM.pdf]

SUPPLEMENTAL DIGITAL CONTENT

SUPPLEMENTAL METHODS

Primary culture of endothelial cells from pulmonary veins

Endothelial cells were cultured as described previously (1). Briefly, normal SD rats were anesthetized as described above. Sodium heparin (400 U, i.p.) was administered to the rats 30 min before surgery. The thoracic cavity was opened, and the heart was removed. Pulmonary veins were obtained and washed six times with phosphate-buffered saline (PBS) in culture dishes. The veins were cut into 5 mm × 5 mm pieces and the inner surface was pasted onto cell culture bottles (Corning, Corning, NY, USA). The veins were then cultured in culture medium (ECM, ScienCell, Carlsbad, CA) containing 10% fetal bovine serum, 1% streptomycin and penicillin, and 1% ECGS (ECM, ScienCell, Carlsbad, CA) for 72 hours. The second passage of pulmonary VECs was used for experiments.

Isolation of neutrophils and platelets from whole blood

The isolation of neutrophils and platelets was performed by using a neutrophil separation kit (TBD Science, Tianjing, China). Briefly, for platelet isolation, whole blood from rats was subjected to centrifugation at 200 g twice, producing platelet-rich plasma, which was then subjected to centrifugation at 1200 g for 15 min. The sedimentary platelets were washed with PBS and then incubated in culture medium for subsequent experiments. For neutrophil isolation, the Ficoll-Hypaque method, which is a density gradient centrifugation method, was used. The whole blood from rats was subjected to centrifugation at 300 g for 20 min. Six percent dextran with a

molecular weight of 500,000 was added to the sedimentary whole blood cells, which were then subjected to thorough suspension and sedimentation for 30 min at room temperature. Leukocyte-rich supernatant was obtained and subjected to centrifugation at 275 g for 6 min. The sediments were resuspended in PBS, and Ficoll-Hypaque (TBD Science, Tianjing, China) with a density of 1.077 was added, and the suspension was then subjected to centrifugation at 750 g at room temperature. The leukocyte- and erythrocyte-rich layer was obtained, mixed with 0.155 M NH_4Cl to lyse the erythrocytes, and washed with 0.25% BSA Hanks buffer for subsequent experiments.

Hypoxia/reoxygenation treatment of endothelial cells, neutrophils and platelets

Endothelial cells, neutrophils and platelets were transferred into a hypoxia culture compartment (MIC-101, Billups-Rothenberg, Del Mar, CA) and equilibrated at 95% N2 and 5% CO2 with an estimated oxygen concentration of less than 0.2% (2). Endothelial cells, neutrophils and platelets were incubated under hypoxic conditions for 4 hours, and then the culture medium was oxygenated for 1 hour. The culture medium was used to isolate MPs according to the methods described above.

Immunolabeling of MPs

PKH26 (Sigma, USA), a red fluorescence dye that binds to the lipid bilayer, was used to label the MPs (3). The MPs were labeled with PKH26 as described in the manufacturer’s instructions. Briefly, the MPs, including EMPs, NMPs, PMPs and plasma MPs, were resuspended in 0.5 mL of diluent C and then quickly mixed with

0.5 mL of diluent C containing 1 μL of PKH26 for 1 min at room temperature. Then, 1 mL FBS was added to stop the reaction, and 5 mL serum-free medium was added to wash away the unbound PKH26 through centrifugation at 20000 g for 40 min. The PKH26-labeled MPs were used for further experiments.

Transfection of double-stranded RNA (dsRNA)

According to the RNAi instructions, pulmonary vein VECs (1×10^5) at the second passage were inoculated into the six-well cell culture plate and incubated in culture medium containing 10% FBS without antibiotics. When the cell confluence reached ≈70%, the culture medium was replaced with ECM (without FBS or antibiotics), and the cells were incubated for 12 hours. Then, 500 μL ECM (without FBS or antibiotics) and 200 pmol dsRNA (Gene Pharma, Suzhou, China) targeting miR-1, miR-29, miR-126, miR-155, miR-542 were mixed, and 10 μL Lipofectamine 2000 reagent (Invitrogen, Carlsbad, CA, USA) was diluted with 500 μL DMEM (without FBS and antibiotics). The diluted dsRNA and RNAi-mate were mixed to form a dsRNA/lipofectamine complex. One milliliter of dsRNA/lipofectamine was added to the six-well cell culture plate and incubated for 24 hours.

Measurement of the permeability of the pulmonary vasculature and endothelial cell monolayers

The Evans blue dye extravasation method for measuring the permeability of the pulmonary vascular endothelium was performed. The Evans blue solution (0.1 mg/mL) was intravenously administered at a dose of 0.5 mg/kg 5 min before surgery. Then, the windpipe was exposed and ligated when the inhalation reached a maximum. A

physiological saline solution was used to wash the lung through cardiopulmonary cycle until the lung became white. The lung was photographed to show dye leakage. The TER values and infiltration rate of endothelial cell monolayers were performed as described previously (1).

Histological analysis and immunofluorescence

After windpipe ligation, the lung was lavaged with oxygenated saline and then fixed with 4% neutral-buffered paraformaldehyde through the right ventricle-lung-left atrium pathway. Then, the lung was fixed in 4% neutral-buffered paraformaldehyde for 48-72 hours. The lung was sectioned at a thickness of 0.5 cm and then subjected to hematoxylin-eosin (HE) staining. An inverted fluorescence microscope (Leica, Germany) was used for histological analysis. For endothelial cell immunofluorescence, the process was performed as described previously (4).

Mesentery microvessel immunofluorescence was also performed. The laparotomy exposed the microvessels, which were permeabilized using 0.25% Triton X-100 in PBS (30 min, room temp), blocked in 3% BSA in TBS-T (30 min, room temp), and probed with primary antibodies against CD31 (Invitrogen, Carlsbad, CA, USA) (to stain the endothelium), occludin (Invitrogen, USA), VE-Cadherin (Invitrogen, USA), ZO-1 (Boster, Wuhan, China), Claudin-5 (Invitrogen, USA) and caveolin-1 (Santa Cruz, USA) and appropriate secondary antibodies conjugated with Alexa-546 or -488 (Invitrogen, USA) for 1 hour at 37 ℃ before finally being mounted on slides. The slides were analyzed using a laser confocal scanning microscope (Leica, Germany).

Transmission electron microscopy

Briefly, MPs were suspended in 100 μL PBS and fixed with 4% glutaraldehyde in 0.1 M phosphate buffer (pH 7.4) at room temperature for 30 min. A drop of each MP sample was placed on a carbon-coated copper grid and immersed in 2% phosphotungstic acid solution (pH 7.0) for 30 sec. The preparations were examined with a transmission electron microscope (JEM-1200EX; JEOL Ltd., Tokyo, Japan) at an acceleration voltage of 80 kV.

Luciferase reporter assays

The psiCHECK-2 vector containing wild-type and mutant ZO-1 3′ UTR and miR-155 mimics were purchased from GeneCopoeia Inc. (USA). The PsiCHECK-2 vector was used as a blank control, and 293T cells were used. Briefly, 500 μL DMEM (without FBS or antibiotics) and 200 pmol miR-155 mimics were mixed, and 10 μL Lipofectamine 2000 reagent (Invitrogen, USA) was diluted with 500 μL DMEM. The diluted miR-155 mimics and Lipofectamine 2000 reagent were mixed and added to a six-well cell culture plate and incubated for 24 hours. The luciferase reporter plasmid was cotransfected into 293T cells. Luciferase activity was detected with a dual-luciferase reporter assay system (Promega, USA).

Statistical analysis

The data are the mean ± standard deviation of n observations. Differences between experimental groups were analyzed by one-way or two-way analysis of variance and Tukey’s post hoc test. A value of $P<0.05$ was considered significant.

REFERENCES

1. Zhang J, Yang GM, Zhu Y, et al: Role of connexin 43 in vascular hyperpermeability and relationship to Rock1-MLC20 pathway in septic rats. Am J Physiol Lung Cell Mol Physiol 2015; 309(11):1323-1232

2. Webster KA, Discher DJ, Bishopric NH: Induction and nuclear accumulation of fos and jun proto-oncogenes in hypoxic cardiac myocytes. J Biol Chem 1993; 268(22):16852-16858

3. Wang Y, Zhang L, Li Y, et al: Exosomes/microvesicles from induced pluripotent stem cells deliver cardioprotective miRNAs and prevent cardiomyocyte apoptosis in the ischemic myocardium. Int J Cardiol 2015; 192:61-69.

4. Zhang J, Yang G, Zhu Y, et al: Relationship of Cx43 regulation of vascular permeability to osteopontin-tight junction protein pathway after sepsis in rats. Am J Physiol Regul Integr Comp Physiol 2018; 314(1):R1-R11.

SUPPLEMENTAL FIGURES

Supplemental Figure 1. Changes of vascular permeability and MPs production after I/R, related to Figure 1.

A: Identification of MPs in circulation by TEM. **B**: Statistical analysis of **Fig 1D** (n=4). **C**: The protein concentration in bronchoalveolar lavage fluid (BALF) (n=8). **D**: MP production in VECs after 30 min of hypoxia/60 min of reoxygenation. **E**: The change in TER of endothelial cell monolayers after H/R treatment (n=8). **F** : The effects of intravenous injection of NC-MPs and I/R-MPs on pulmonary vascular permeability, as measured by the leakage of FITC-labeled albumin (n=8). **G:** The effect of NC-MPs and I/R-MPs on mesenteric microvascular permeability, as measured by the leakage of FITC-labeled albumin. **H:** The effect of NC-MPs and I/R-MPs on the permeability of pulmonary VEC monolayers as measured by normalized TER (n=8). **I**: Statistical analysis of **Fig 1F** (n=4). **J:** The protein concentration in BALF 2 hours after intravenous injection of NC-MPs and I/R-MPs (n=8). The data are the mean ± SD of n experiments. **N:** normal group; **Sham:** sham-operated group; **I/R:** ischemia/reperfusion group; **H/R:** Hypoxia/Reoxygenation group. **NC-MP**: MPs derived from the blood of normal rats. **I/R-MP**: MPs derived from the blood of ischemia/reperfusion rats. **P<0.01 vs. N or sham group.

Supplemental Figure 2. The combined effects of EMPs and PMPs in regulation of vascular permeability, related to Figure 2.

A: The changes in EMPs, PMPs and NMPs in the culture medium of VECs, platelets and neutrophils, respectively, after H/R treatment. **B**: The effect of

H/R-EMPs, H/R-PMPs and H/R-NMPs on leakage of FITC-labeled albumin from the microvasculature. **C**: The changes in the TER of VEC monolayers after stimulation with H/R-EMPs, H/R-PMPs and H/R-NMPs. **D**: The effects of H/R-EMPs, H/R-PMPs and H/R-NMPs on the protein concentration in BALF. **E, F:** The effects of H/R-EMPs on the concentrations of EMPs, PMPs and NMPs in plasma (**E**) and BALF (**F**). **G, H**: The effects of H/R-PMPs on concentrations of EMPs, PMPs and NMPs in plasma (**G**) and BALF (**H**). **I, J**: The effects of H/R-NMPs on concentrations of EMPs, PMPs and NMPs in plasma (**I**) and BALF (**J**). **K**: The effects of H/R-EMPs, H/R-PMPs and H/R-PMPs on pulmonary sequestration of platelets as shown by immunohistochemical staining with CD41 antibodies. The data are the mean ± SD of n experiments (n>3). **VEC**: vascular endothelial cell; **PLT**: platelet; **NeutP**: neutrophil; **NC-NMP**, **NC-EMP, NC-PMP**: NMPs, EMPs and PMPs derived from normal neutrophils, endothelial cells and platelets, respectively; **H/R-NMP**, **H/R-EMP**, **H/R-PMP**: NMPs, EMPs and PMPs derived from hypoxia/reoxygenation-treated neutrophils, endothelial cells and platelets, respectively. **P<0.01 vs. N or sham group.

Supplemental Figure 3. Location of MPs after intravenously injection, related to Figure 3.

A: The mesenteric microvascular location of PKH26 (red)-labeled H/R-NMPs, H/R-EMPs, and H/R-PMPs after intravenous administration. **B**: Localization of PKH26 (red)-labeled MPs in VECs (green) after 1-hour of coculture and three washes with PBS. **C**: The inhibition and overexpression effects of double strand RNA and

mimic RNA. **D**: The EMPs produced by pulmonary VECs transfected with red fluorescence miR-155 transported miR-155 (red) to pulmonary VECs. The data are the mean ± SD of n experiments (n>4). **Veh:** negative control; **Cal-AM**: Calcein AM, which was used to profile cells; **Neg-Ctl**: negative control; *P<0.05, **P<0.01 vs N group.

Supplemental Figure 4. The effects of miR-155 and miR-126 in H/R-EMP- and H/R-PMP on expression of proteins association with vascular permeability, related to Figure 4.

A: Statistical analysis of Fig 4A. **B**: Statistical analysis of Fig 4C. **C-E**: The effect of H/R-EMP and H/R-PMP on expression of proteins and tight junction structure association with vascular permeability in pulmonary VECs. **F**: Statistical analysis of Fig 4D. **G**: Statistical analysis of Fig 4E. The data are the mean ± SD of n experiments (n>3). *P<0.05 vs normal group. **P<0.01 vs normal group.
