# Supplementary figures and images for: Synergistic effects of EMPs and PMPs on pulmonary vascular leakage and lung injury after ischemia/reperfusion
Source: Cell Commun Signal. 2020 Nov 23;18:184. doi: 10.1186/s12964-020-00672-0 (PMC7682096; doi:10.1186/s12964-020-00672-0)

## Slide 1
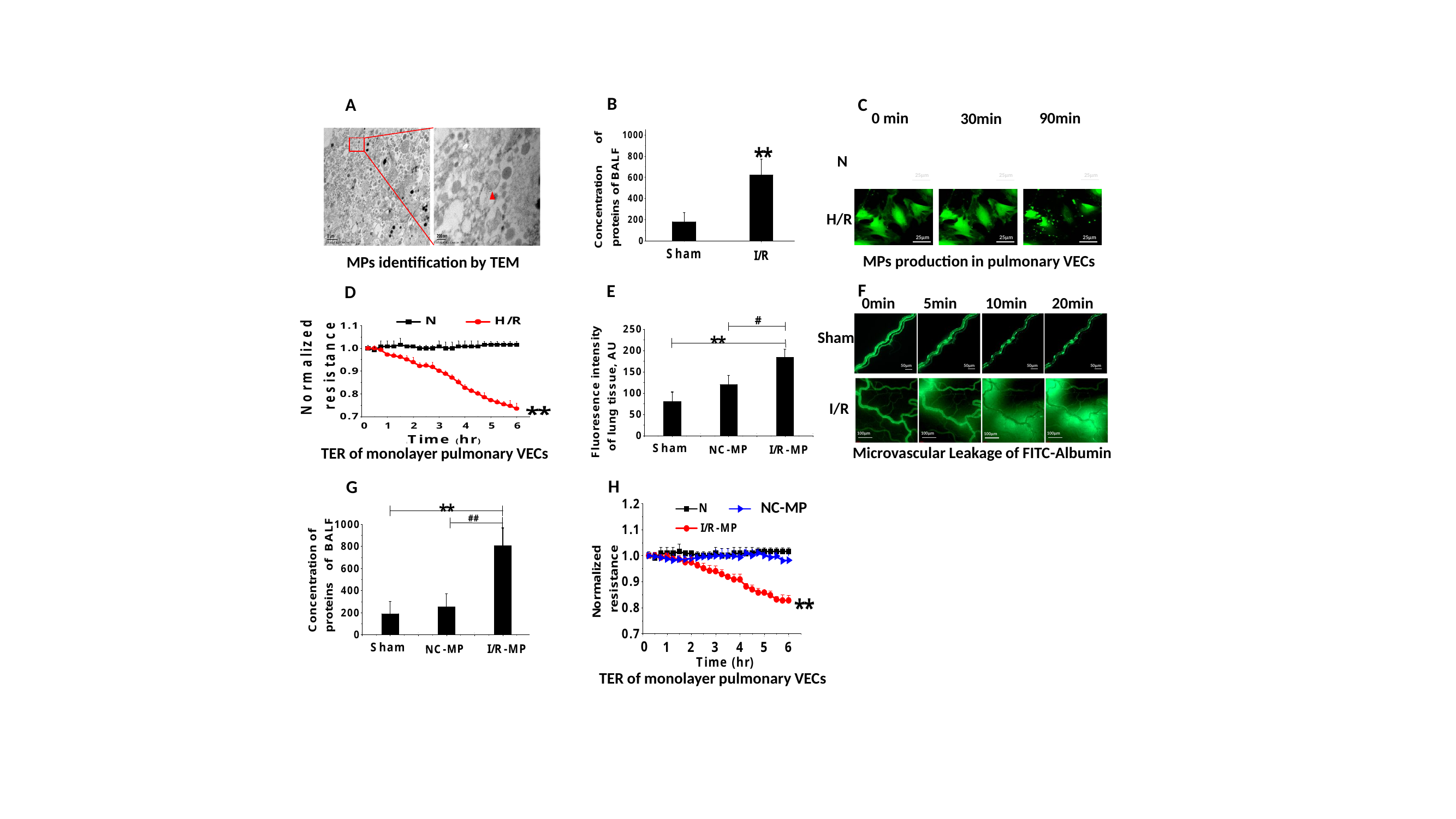

Supplement: Supplementary file 3 — Additional file 2. Supplemental Figure 1. [file 12964_2020_672_MOESM3_ESM.pptx]

## Slide 1
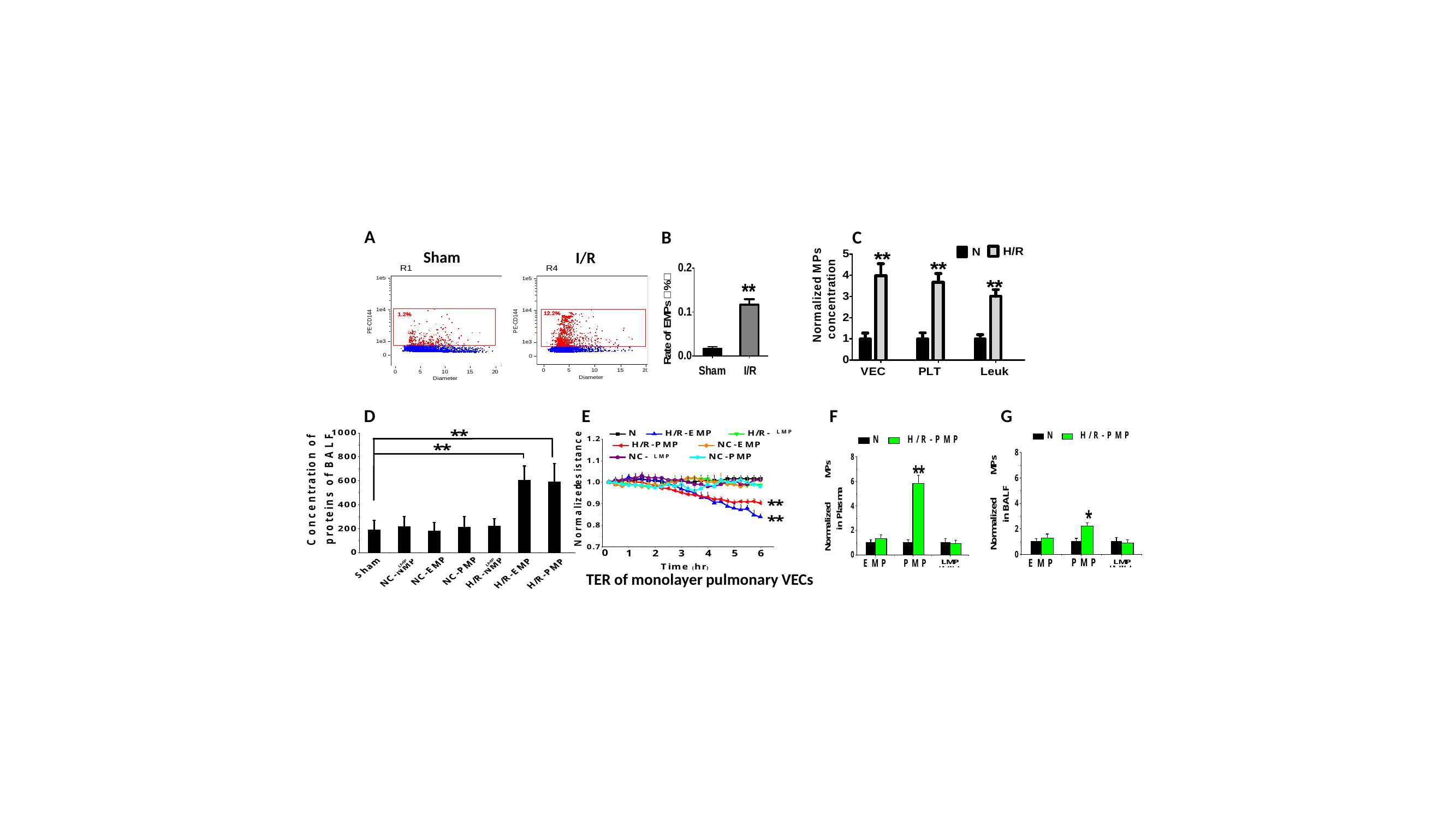

Supplement: Supplementary file 4 — Additional file 3. Supplemental Figure 2. [file 12964_2020_672_MOESM4_ESM.pptx]

## Slide 1
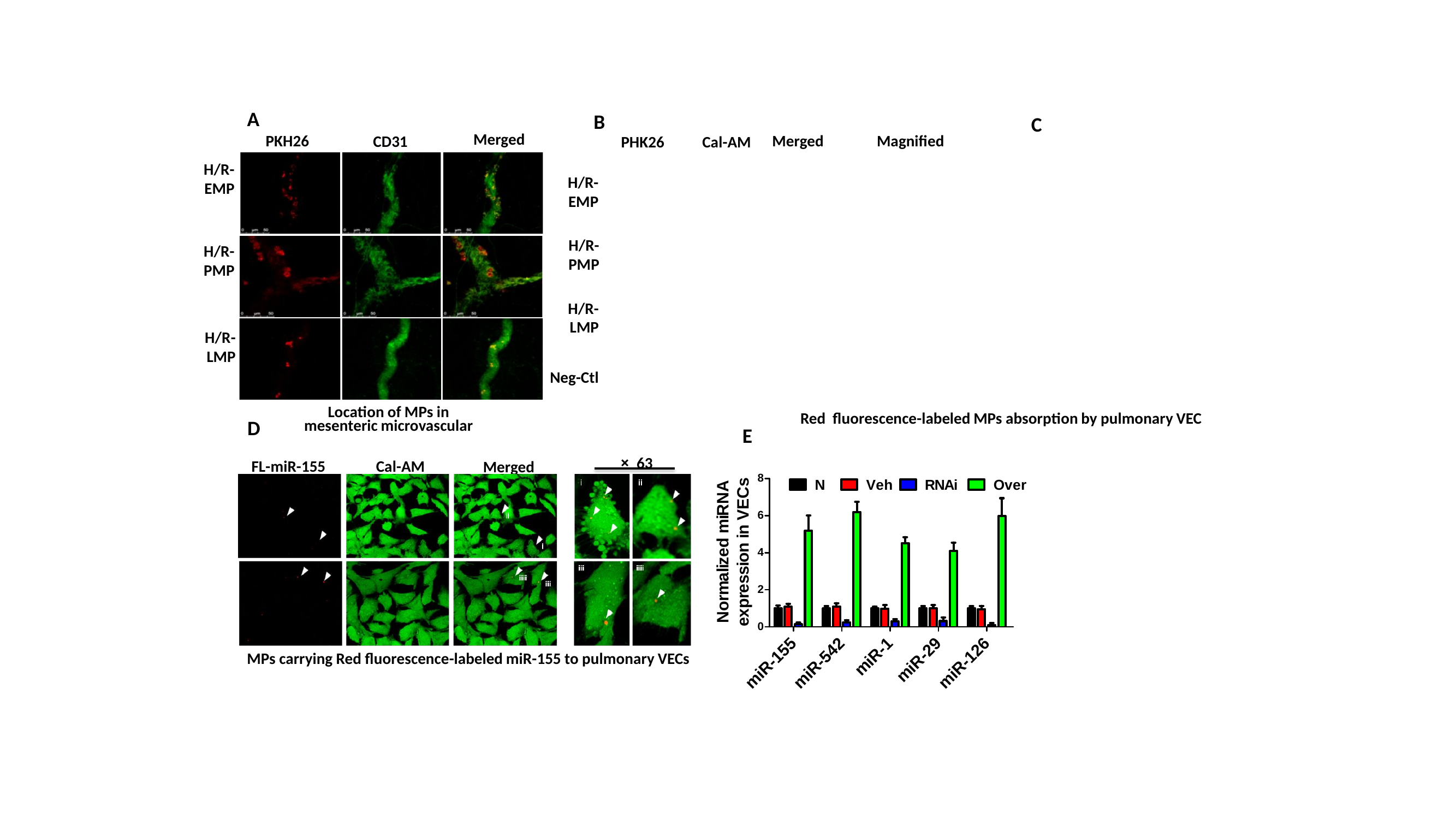

Supplement: Supplementary file 5 — Additional file 4. Supplemental Figure 3. [file 12964_2020_672_MOESM5_ESM.pptx]

## Slide 1
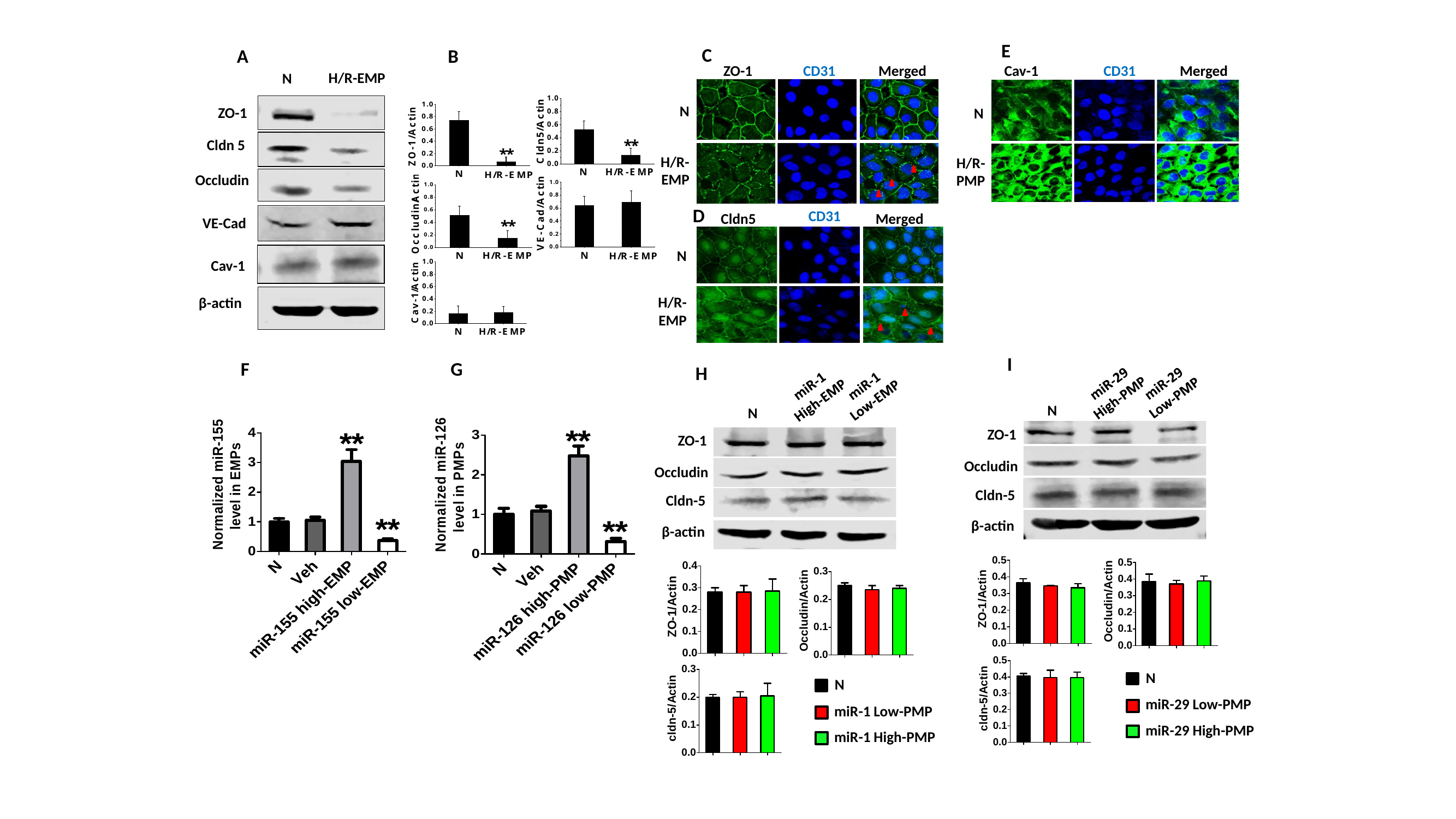

Supplement: Supplementary file 6 — Additional file 5. Supplemental Figure 4. [file 12964_2020_672_MOESM6_ESM.pptx]
